# Prevalence of actionable mutations and copy number alterations and the price of a genomic testing panel

**DOI:** 10.18632/oncotarget.11994

**Published:** 2016-09-13

**Authors:** Chan Shen, Funda Meric-Bernstam, Xiaoping Su, John Mendelsohn, Sharon Giordano

**Affiliations:** ^1^ Department of Health Services Research, The University of Texas MD Anderson Cancer Center, Houston, TX, USA; ^2^ Department of Biostatistics, The University of Texas MD Anderson Cancer Center, Houston, TX, USA; ^3^ Departments of Investigational Cancer Therapeutics and Surgical Oncology, The University of Texas MD Anderson Cancer Center, Houston, TX, USA; ^4^ Departments of Bioinformatics and Computational Biology, The University of Texas MD Anderson Cancer Center, Houston, TX, USA; ^5^ Department of Genomic Medicine, The University of Texas MD Anderson Cancer Center, Houston, TX, USA; ^6^ Center for Health and Biosciences, Baker Institute, Rice University, Houston, TX, USA

**Keywords:** genomic testing panel, cancer, costs, mutations, copy number alterations

## Abstract

Interest in genomic testing for the selection of cancer therapy is growing. However, the cost of genomic testing has not been well studied. We sought to determine the price of identifying mutations and copy number alterations (CNAs) in theoretically actionable genes across multiple tumor types. We reviewed data from The Cancer Genome Atlas to determine the frequency of alterations in nine tumor types. We used price information from a commonly used commercial genomic testing platform (FoundationOne) to determine the price of detecting mutations and CNAs in different types of tumors. Although there are large variations in the prevalence by tumor type, when the detection of both mutations and CNAs was considered overall, most patients had at least one alteration in a potentially actionable gene (84% overall, range 51%- 98% among tumor types assessed). The corresponding average price of identifying at least one alteration per patient ranges from $5,897 to $11,572. Although the frequency of mutations and CNAs in actionable genes differs by tumor type, most patients have an actionable genomic alteration detectable by a commercially available panel. Determining CNAs as well as mutations improves actionability and reduces the price of detecting an alteration.

## INTRODUCTION

Genomic medicine is a rapidly growing field in oncology. In the past decade, we have seen growth in the number of new genomic tests available, and genomic testing is now often used to match patients to approved or investigational agents. However, genomic testing is expensive and may not be covered by insurance providers; thus, it can pose a significant financial burden on cancer patients. Currently, the literature on the cost of genomic testing in patients with cancer is limited. Although there are a few studies on the cost-effectiveness of genomic testing panel for specific cancer and subpopulation [1, 2], the comparison of prices of detection between different cancer types is largely unknown. Herein, we evaluate the prevalence of genomic alterations, the likelihood of detecting mutations and copy number alterations (CNAs) in actionable genes, and the relevant prices for detecting these alterations in several cancer types. This study aims to help researchers and practitioners understand the costs of identifying theoretically actionable alterations in multiple tumor types.

## RESULTS

Table 1 shows the prevalence of testable mutations that were theoretically or pharmaceutically actionable and the average price for identifying one patient with mutations in actionable genes by cancer type. Among 986 breast cancer patients in TCGA data, 586 (59%) had mutations in genes that were theoretically actionable and tested in the FoundationOne test. The frequency of mutations in theoretically actionable genes ranged from 25% in ovarian cancer to 93% in endometrial cancer. The price ranged from $22,907 in ovarian cancer to $6,254 in endometrial cancer. The prevalence of testable mutations in pharmaceutically actionable genes is relatively lower. The frequency of mutations in pharmaceutically actionable genes ranged from 10% in ovarian cancer to 72% in endometrial cancer, with corresponding price ranging from $55,556 in ovarian cancer to $8,035 in endometrial cancer.

**Table 1:** Prevalence of Actionable Mutations by Cancer Type

cancer type	Total number of patients	Theoretically Actionable			Pharmaceutically Actionable		
		Frequency	Percentage	Cost/case*	Frequency	Percentage	Cost/case*
breast	986	586	59.4	$9,759	388	39.4	$14,740
colon adenocarcinoma	154	129	83.8	$6,924	105	68.2	$8,507
lung adenocarcinoma	248	205	82.7	$7,017	163	65.7	$8,824
lung squamous cell carcinoma	178	150	84.3	$6,883	95	53.4	$10,868
ovarian	316	80	25.3	$22,907	33	10.4	$55,556
glioblastoma multiforme	283	225	79.5	$7,295	129	45.6	$12,725
endometrial cancer	248	230	92.7	$6,254	179	72.2	$8,035
kidney clear cell carcinoma	491	234	47.7	$12,170	98	20.0	$29,058
head and neck cancer	306	252	82.4	$7,043	132	43.1	$13,445

Table 2 shows the prevalence of testable CNAs that were theoretically or pharmaceutically actionable and the price for identifying at least one actionable CNA. Notably, the rate of CNAs in theoretically actionable genes varied significantly by disease, from 475 (83%) of 571 glioblastoma multiforme patients to 15 (3%) of 504 clear cell renal cell carcinoma patients. Similarly, the prevalence of mutations in pharmaceutically actionable genes also varied substantially from 55% in glioblastoma multiforme patients to 1% in kidney clear cell carcinoma. Table 3 shows the prevalence of testable mutations and CNAs combined. In this table, we considered any patient who had at least one testable mutation or CNA as one actionable case. The table shows a higher prevalence and lower price than the first two tables. The prevalence of theoretically actionable mutations or CNAs was above 80% for all cancer types we studied except clear cell renal cell carcinoma where the prevalence was 50%. Endometrial cancer patients had the highest prevalence of 98%. Accordingly, the price ranged from $5,897 to $11,572 to identify one patient with theoretically actionable alterations. The prevalence of mutations or CNAs pharmaceutically actionable showed a similar pattern.

**Table 2:** Prevalence of Actionable Copy Number Alterations by Cancer Type

cancer type	Total number of patients	Theoretically Actionable			Pharmaceutically Actionable		
		Frequency	Percentage	Cost/case*	Frequency	Percentage	Cost/case*
Breast	1033	535	51.8	$11,199	281	27.2	$21,324
colon adenocarcinoma	427	144	33.7	$17,200	66	15.5	$37,516
lung adenocarcinoma	493	175	35.5	$16,338	67	13.6	$42,678
lung squamous cell carcinoma	489	326	66.7	$8,700	228	46.6	$12,438
Ovarian	569	410	72.1	$8,049	224	39.4	$14,732
glioblastoma multiforme	571	475	83.2	$6,972	316	55.3	$10,481
endometrial cancer	504	132	26.2	$22,146	54	10.7	$54,155
kidney clear cell carcinoma	504	15	3.0	$194,631	5	1.0	$585,859
head and neck cancer	388	204	52.6	$11,031	90	23.2	$25,000

**Table 3:** Prevalence of Either Actionable Mutations or Actionable CNAs by Cancer Type

cancer type	Total number of patients	Theoretically Actionable			Pharmaceutically Actionable		
		Frequency	Percentage	Cost/case*	Frequency	Percentage	Cost/case*
breast	962	791	82.2	$7,054	568	59.0	$9,824
colon adenocarcinoma	152	142	93.4	$6,209	118	77.6	$7,471
lung adenocarcinoma	172	161	93.6	$6,197	132	76.7	$7,558
lung squamous cell carcinoma	178	170	95.5	$6,073	141	79.2	$7,322
ovarian	311	254	81.7	$7,102	140	45.0	$12,883
glioblastoma multiforme	273	266	97.4	$5,952	211	77.3	$7,504
endometrial cancer	242	238	98.4	$5,897	188	77.7	$7,466
kidney clear cell carcinoma	415	208	50.1	$11,572	86	20.7	$27,992
head and neck cancer	302	278	92.1	$6,301	171	56.6	$10,244

* Cost/case indicates the average cost for identifying one patient that has testable and actionable gene(s) based on FoundationOne test list price.

## DISCUSSION

In this study we found significant variations in the prevalence of actionable gene mutations and CNAs among different types of tumors. This finding is in line with previous studies using hot-spot mutation testing platforms [7, 8]. However, for all the cancer types that we considered, the majority of patients had theoretically actionable gene mutations or CNAs that can be detected in one commercially available genomic test panel. In this paper, we focused on the next generation sequencing gene panels and did not consider routine tumor molecular profiling that may involve multiple assessments, each of which targets a single gene or type of mutation (e.g. HER2, BRCA1, BRCA2 in breast cancer, and EGFR, HER2, KRAS, and ALK in lung cancer). Although the price of a single gene test may be lower, it is likely that when traditional methods are used for multiple assessments, a larger quantity of DNA is needed and it leads to longer turnaround time. Given the rapidly growing number of genes tested in genomic test panels, we expect that the proportion of patients with testable and actionable gene mutations or CNAs will continue to grow. The number of targeted therapies has been growing rapidly in recent years. The targeted therapies in use today may cost 10,000 to 25,000 dollars for each treatment given. The genomic testing results can steer physicians and patients towards the experimental treatments that may be effective and away from the treatments that are unlikely to be effective for that patient. Combining the growing number of genes tested in panels with the growing number of expensive targeted drug therapies and the trend of falling prices for genomic tests, genomic testing is poised to become more cost-effective when the entire course of treatment is taken into account.

This study has several limitations. First, the prevalence of gene mutations and CNAs was based on TCGA data, which may not reflect advanced/metastatic disease. Second, mutations may differ in their functional impact, and thus not all mutations in actionable genes are actionable. Third, not all theoretically actionable alterations are actionable in the context of the specific disease or genomic co-alterations. Fourth, KRAS was considered actionable in our analyses, which may inflate the prevalence of actionable genes. Fifth, we focused on mutations and CNAs only, without taking fusions into account; use of assays such as FoundationOne which provide not only mutation and CNA but also fusion information and common fusions, would increase the prevalence of actionable genomes. Finally it is important to recognize that the actual actionability for patients depends heavily on the trial availability [9]. Nevertheless, this is the first study that aims at understanding the costs of identifying actionable alterations using a genomic testing panel.

## MATERIALS AND METHODS

We downloaded the most recent data from The Cancer Genome Atlas (TCGA) via the TCGA Data Portal [3]. TCGA provides data on clinical information, genomic characterization, and high-level sequence analysis of tumor genomes. In this study, we examined both somatic mutations and CNAs for nine cancer types: breast cancer, colon adenocarcinoma, lung adenocarcinoma, lung squamous cell carcinoma, ovarian cancer, glioblastoma multiforme, endometrial cancer, clear cell renal cell carcinoma, and head and neck cancer. For each cancer type, we determined the prevalence of specific somatic mutations and CNAs using TCGA data. Of note the TCGA data included a sample of patients with somatic mutation information, a sample of patients with CNA information and another subsample of patients with both somatic mutation and CNA information. We were not able to identify copy-neutral loss of heterozygosity (LOH) since this type of data was not provided by TCGA analysis group. Notice that we used curated TCGA somatic SNV nutation data instead of pipeline-generated SNV in this study. We only used focal copy number alterations, which were generated by GISTIC analysis. For the prevalence of CNAs, we used conservative thresholds to define copy number amplification and deletion. More specifically, if the copy number was above 6, the patient was considered to have copy number amplification, and if the copy number was below 1, the patient was considered to have copy number deletion; otherwise, the patient was considered to have non-significant CNAs. Such cutoffs are in line with reporting thresholds for next generation sequencing gene panels such as FoundationOne testing, on which we focus in our price calculation.

After establishing the prevalence of mutations and CNAs for the different cancer types that we studied, we matched it with the list of mutations that are testable in FoundationOne to obtain the prevalence of “testable” mutations and CNAs. We chose FoundationOne because it is a commonly used commercial genomic testing panel and because it is the only genomic testing panel currently available on the market with clear information on price and the list of genes that are covered in the test panel [4]. Starting from this testable list, we established the prevalence of “actionable” mutations and CNAs so as to arrive at a list that was both testable and actionable. Here, we distinguished between amplification and deletion for CNAs. Genes were determined as theoretically actionable if a FDA-approved or clinically available investigational drug either directly or indirectly targets the gene, as previously described [5]. For each gene under consideration, public Web sites (NCI Drug Dictionary, NCI Thesaurus, Selleckchem, Medkoo, DGIdb, PubMed, and ClinicalTrials.gov) were consulted to identify drugs that target the encoded protein at clinically relevant IC50 values, as determined experimentally. PubMed was used to search for relevant literature that demonstrated either preclinical or clinical sensitivity of the drug to genetic alterations in the gene of interest. Drugs targeting proteins downstream of the gene of interest (indirect targets) were also identified in this manner with corroborating published literature indicating their sensitivity to genetic alterations in the gene of interest. Potentially actionable genes are listed in Table 4. As the impact of genomic analysis on therapeutic decisions may differ depending on specific genes, we have also included a table (Table 5) that shows the five most frequently observed mutations for each tumor type to allow researchers to best assess the prevalence of actionable genes.

**Table 4:** Therapeutic implications of potentially actionable genes

Gene	Potential therapeutic implications	Actionability		
		Mutations	CNAs	
			Amplification	Deletion
ABL1	Treatment with ABL or BCR-ABL inhibitors	Yes	Yes	No
ABL2	Treatment with ABL inhibitors	Yes	Yes	No
AKT1	Treatment with AKT or mTOR inhibitors	Yes	Yes	No
AKT2	Treatment with AKT or mTOR inhibitors	Yes	Yes	No
AKT3	Treatment with AKT or mTOR inhibitors	Yes	Yes	No
ALK	Treatment with ALK inhibitors	Yes	Yes	No
AR*	Resistance to anti-hormone therapy	Yes	Yes	No
ARAF	Treatment with RAF inhibitor	Yes	No	No
ATM	Treatment with PARP inhibitors	Yes	No	Yes
ATR	Treatment with PARP inhibitors	Yes	No	Yes
AURKA	Treatment with AURKA inhibitors	Yes	Yes	No
AURKB	Treatment with AURKB inhibitors	Yes	Yes	No
BAP1	Treatment with HDAC inhibitors	Yes	No	Yes
BCL2	Treatment with BCL2 inhibitor and potential resistance to mTOR inhibitors Resistance to BCL2 inhibitor	Yes	Yes	No
BRAF	Treatment with BRAF inhibitors	Yes	Yes	No
BRCA1	Treatment with PARP inhibitors	Yes	No	Yes
BRCA2	Treatment with PARP inhibitors	Yes	No	Yes
CCND1	Treatment with CDK 4/6 inhibitors	Yes	Yes	No
CCND2	Treatment with CDK 4/6 inhibitors	Yes	Yes	No
CCND3	Treatment with CDK 4/6 inhibitors	Yes	Yes	No
CCNE1	Treatment with CDK 2 Inhibitors	Yes	Yes	No
CDK4	Treatment with CDK 4/6 inhibitors	Yes	Yes	No
CDK6	Treatment with CDK 4/6 inhibitors	Yes	Yes	No
CDKN1B	Treatment with CDK 2 Inhibitors	Yes	No	Yes
CDKN2A	Treatment with CDK 4/6 inhibitors	Yes	No	Yes
CDKN2B	Treatment with CDK 4/6 inhibitors	Yes	No	Yes
CDKN2C	Treatment with CDK 4/6 inhibitors	Yes	No	Yes
CHEK2	Treatment with Chk2 inhibitor	Yes	Yes	No
CSF1R	Treatment with CSF1R monoclonal antibody and inhibitors	Yes	Yes	No
DDR2	Treatment with DDR2 inhibitor	Yes	Yes	No
DNMT3A	High risk” factor of myelodysplastic or myeloproliferative disorders required for trial enrollment.	Yes	No	No
DOT1L	Treatment with DOT1L inhibitor	Yes	Yes	No
EGFR	Treatment with EGFR inhibitors	Yes	Yes	No
EPHA3*	Treatment with Dasatinib	Yes	Yes	No
ERBB2 (HER2)	Treatment with HER2 inhibitors, monoclonal antibodies, and targeted vaccines	Yes	Yes	No
ERBB3 (HER3)	Treatment with HER3 inhibitors	Yes	Yes	No
ERBB4 (HER4)	Treatment with HER4 inhibitors	Yes	Yes	No
ESR1	Anti-hormone resistance	Yes	No	No
FGF10	Trial enrollment	Yes	Yes	No
FGF14				
FGF19				
FGF23				
FGF3				
FGF4				
FGF6				
FGFR1	Treatment with FGFR1 inhibitors	Yes	Yes	No
FGFR2	Treatment with FGFR2 inhibitors	Yes	Yes	No
FGFR3	Treatment with FGFR3 inhibitors	Yes	Yes	No
FGFR4	Treatment with FGFR4 inhibitors	Yes	Yes	No
FLT1	Treatment with FLT1 inhibitors	Yes	Yes	No
FLT4	Treatment with FLT4 inhibitors	Yes	Yes	No
GNA11	Treatment with PKC and MEK inhibitors	Yes	Yes	No
GNAQ	Treatment with PKC and MEK inhibitors	Yes	Yes	No
HGF	Treatment HGF monoclonal antibody	Yes	Yes	No
HRAS	Treatment with MEK Inhibitors	Yes	Yes	No
IGF1R	Treatment with IGF1R monoclonal antibodies or inhibitors	Yes	Yes	No
IGF2	Treatment with IGF1R monoclonal antibodies or inhibitors	Yes	Yes	No
JAK1	Treatment with JAK inhibitors	Yes	Yes	No
JAK2	Treatment with JAK inhibitors	Yes	Yes	No
JAK3	Treatment with JAK inhibitors	Yes	Yes	No
KDR	Treatment with KDR inhibitors	Yes	Yes	No
KIT	Treatment with KIT inhibitors	Yes	Yes	No
KRAS	Treatment with MEK Inhibitors	Yes	Yes	No
MAP2K1	Treatment with MEK Inhibitors	Yes	Yes	No
MAP2K2	Treatment with MEK Inhibitors	Yes	Yes	No
MAP2K4	Treatment with JNK1 inhibitor	Yes	Yes	No
MAP3K1	Treatment with JNK1 inhibitor	Yes	Yes	No
MDM2	Treatment with MDM2 inhibitor or Nutlins that inhibit MDM2-p53 interaction.	Yes	Yes	No
MET	Treatment with MET inhibitors (Crizotinib, Cabozantinib)	Yes	Yes	No
MPL*	Treatment with JAK2 inhibitors.	Yes	No	No
MTOR	Treatment with mTOR inhibitors	Yes	Yes	No
MYCN	Treatment with BET inhibitors	Yes	Yes	No
NF1	Treatment with PI3K pathway inhibitors (PI3K/AKT/MTOR), MAPK pathway inhibitors (RAF/MEK/ERK), or HSP90 inhibitors	Yes	No	Yes
NF2	Treatment with PI3K pathway inhibitors (PI3K/AKT/MTOR), MAPK pathway inhibitors (RAF/MEK/ERK), or HSP90 inhibitors	Yes	No	Yes
NOTCH1	Treatment with Gamma Secretase inhibitors (GSIs)	Yes	Yes	No
NOTCH2	Treatment with GSIs Resistance to GSIs	Yes	Yes	No
NOTCH3	Treatment with GSIs	Yes	Yes	No
NPM1	Correlate with positive response to all-trans retinoic acid therapy and chemotherapy in AML.	Yes	No	No
NRAS	Treatment with MEK inhibitors	Yes	Yes	No
NTRK1	Treatment with NTRK1 (TrkA) inhibitor	Yes	Yes	No
NTRK2	Treatment with NTRK2 (TrkB) inhibitor	Yes	Yes	No
NTRK3	Treatment with NTRK3 (TrkC) inhibitor	Yes	Yes	No
PDGFRA	Treatment with PDGFRA inhibitors	Yes	Yes	No
PDGFRB	Treatment with PDGFRB inhibitors	Yes	Yes	No
PIK3CA	Treatment with PI3K, AKT, or mTOR inhibitors	Yes	Yes	No
PIK3CB	Treatment with PIK3CB inhibitors	Yes	Yes	No
PIK3R1	Treatment with PI3K, AKT or mTOR inhibitors	Yes	No	No
PIK3R2	Trial selecting for mutations	Yes	No	No
PTCH1	Treatment with SMO inhibitors	Yes	No	Yes
PTEN	Treatment with p110beta, AKT, or mTOR inhibitors	Yes	No	Yes
PTPN11	Treatment with MEK Inhibitors	Yes	Yes	No
RAD50	Treatment with PARP inhibitors	Yes	No	Yes
RAF1	Potential resistance to RAF inhibitors Treatment with MEK inhibitors Resistance to Dasatinib	Yes	Yes	Yes
RET	Treatment with Ret inhibitors	Yes	Yes	No
SMO	Treatment with SMO inhibitors	Yes	Yes	No
SRC	Treatment with SRC inhibitors	Yes	Yes	No
STK11	Treatment with mTOR or AMPK inhibitors	Yes	No	Yes
SYK	Treatment with Syk inhibitors	Yes	Yes	No
TOP2A*	Treatment with topoisomerase 2A inhibitors	Yes	Yes	Yes
TSC1	Treatment with mTOR inhibitors	Yes	No	Yes
TSC2	Treatment with mTOR inhibitors	Yes	No	Yes

Note. Genes were determined as theoretically actionable if there is an FDA-approved or clinically available investigational drug that either directly or indirectly targets the gene as previously described.

* Borderline classification as actionable.

**Table 5:** Top five most common mutations by cancer type

Cancer type	Gene	Percentage
breast	PIK3CA	32.05%
	MAP3K1	7.10%
	PTEN	3.55%
	MAP2K4	3.25%
	NF1	2.74%
colon adenocarcinoma	KRAS	37.66%
	PIK3CA	16.88%
	ATM	13.64%
	BRAF	12.99%
	NRAS	9.74%
Lung adenocarcinoma	KRAS	24.19%
	NF1	11.29%
	EGFR	10.89%
	KDR	10.48%
	HGF	10.08%
Lung squamous cell carcinoma	PIK3CA	15.17%
	CDKN2A	14.61%
	NF1	11.80%
	NOTCH1	7.87%
	PTEN	7.87%
Ovarian	NF1	2.53%
	BRCA1	2.22%
	BRCA2	2.22%
	EGFR	1.90%
	KIT	1.58%
Glioblastoma multiforme	PTEN	30.74%
	EGFR	26.15%
	PIK3R1	11.31%
	PIK3CA	10.60%
	NF1	10.25%
Endometrial cancer	PTEN	64.92%
	PIK3CA	53.23%
	PIK3R1	33.47%
	KRAS	21.37%
	FGFR2	12.50%
Kidney clear cell carcinoma	BAP1	8.55%
	MTOR	5.09%
	PTEN	3.67%
	ATM	2.44%
	PIK3CA	2.44%
Head and neck cancer	CDKN2A	21.57%
	PIK3CA	20.92%
	NOTCH1	19.28%
	ATR	5.88%
	NOTCH2	5.23%

Further, we examined a smaller list of “pharmaceutically actionable” genes as this is important for the clinical implementation of biomarker-based therapy [5]. These included genes that have already been linked to FDA-approval of a drug (e.g. BRAF inhibitors), and gene variants known to affect drug effectiveness or toxicity, and that affect dosing guidelines and/or drug label information. This list is derived from genes that have well-known pharmacogenomics associations with drugs available on the market based on the Pharmacogenomics Knowledgebase [6]. We provided the list of pharmaceutically actionable genes with the corresponding drugs in the Supplementary Table S1.

Finally, we calculated the average price of identifying one patient with actionable alterations, using the list price of FoundationOne ($5,800) divided by the proportion of patients with at least one actionable alteration. Of note, we focused on the price of detecting “actionable patients”. If the patient had more than one mutation, he/she would still be counted as one. By doing this, we avoided the problem of overestimating the number of patients detected for actionable genes.

The Institutional Review Board at The University of Texas MD Anderson Cancer Center approved this study and waived the requirement for patient consent.

## SUPPLEMENTARY TABLE


