# Supplementary material for: Prevalence of actionable mutations and copy number alterations and the price of a genomic testing panel
Source: Oncotarget. 2016 Sep 13;7(44):71686–95. doi: 10.18632/oncotarget.11994 (PMC5342111; doi:10.18632/oncotarget.11994)
Supplement: Supplementary file 1 [file oncotarget-07-71686-s001.pdf]

Prevalence of actionable mutations and copy number alterations and the price of a genomic testing panel

SUPPLEMENTARY TABLE

Supplementary Table S1: List of Pharmaceutically Actionable Genes with the Corresponding Drugs

Gene	Corresponding Drugs						
ABL1	bosutinib	busulfan	dasatinib	homoharringtonine	imatinib	nilotinib	ponatinib
AKT1	everolimus						
ALK	crizotinib						
BRAF	dabrafenib	trametinib	vemurafenib				
EGFR	afatinib	cetuximab	erlotinib	gefitiniib	panitumumab	regorafenib	
ERBB2	everolimus	lapatinib	pertuzumab	trastuzumab			
ESR1	anastrozole	everolimus	exemestane	fulvestrant	letrozole	tamoxifen	
FGFR1	pazopanib						
FGFR3	pazopanib						
FLT1	pazopanib	regorafenib					
FLT4	pazopanib	regorafenib					
KDR	pazopanib	regorafenib	vandetanib				
KIT	imatinib						
KRAS	cetuximab	panitumumab	regorafenib				
MAP2K1	trametinib						
MAP2K2	trametinib						
MTOR	everolimus						
PDGFRA	pazopanib						
PDGFRB	imatinib						
PIK3CA	everolimus						
RARA	arsenic trioxide	tretinoin					
RET	vandetanib						
TSC1	everolimus						
TSC2	everolimus						
